# A Whole Medical Staff Anæstheticised

**Published:** 1848-04

**Authors:** 


### A WHOLE MEDICAL STAFF ANÆSTHETICISED.

The London *Medical Times* relates the following amusing incident:

At the Taunton Hospital, as a patient was undergoing amputation of a limb, while under the influence of Chloroform, the nurse let fall the bottle containing the Chloroform, which quickly spread its somniferous influence over the operators, and sometime elapsed before they recovered from their partial insensibility.

